# Is the Early Screening of Lower Genital Tract Infections Useful in Preventing Adverse Obstetrical Outcomes in Twin Pregnancy?

**DOI:** 10.3390/jcm13092673

**Published:** 2024-05-02

**Authors:** Sofia Roero, Giulia Benedetto, Lorena Charrier, Agata Ingala, Alice Ronco, Teresa Fea, Valentina Borgarello, Carlotta Bossotti, Silvana Arduino, Alberto Revelli

**Affiliations:** 1 Twin Pregnancy Care Unit, Gynecology and Obstetrics 2U, A.O.U. Città della Salute e della Scienza, Sant’Anna Hospital, University of Turin (Department of Surgical Sciences), Via Ventimiglia 1, 10126 Turin, Italy; 2 Departement of Public Health and Pediatrics, A.O.U. Città della Salute e della Scienza, University of Turin, Via Santena 5, 10126 Turin, Italy

**Keywords:** twin pregnancy, preterm birth, pPROM, lower genital tract infections

## Abstract

**Objectives**: Twin pregnancy implies a higher risk of preterm birth and, consequently, higher neonatal morbidity and mortality. In singleton pregnancies, infections of the lower genital tract (LGTIs) and bacterial vaginosis are associated with preterm labor, and their early detection has been proven effective in reducing complications like the preterm premature rupture of membranes (pPROM) and preterm delivery. The same evidence, however, is lacking for twin pregnancies. This study aimed to evaluate whether the early identification and treatment of LGTIs or bacterial vaginosis in asymptomatic women with twin pregnancy could reduce the rate of miscarriages, pPROM, and preterm birth. **Methods**: This study performed a retrospective comparison of 285 women with a multiple pregnancy submitted for a cervico-vaginal swab only at 20–22 weeks (Single Test Group, STG), and 199 women who underwent the swab at 12–14 and again at 20–22 weeks (Double Test Group, DTG). All women included in the study had a twin pregnancy and were followed up at Sant’Anna Hospital, Turin (Italy), between September 2012 and February 2021. **Results**: In STG, 21.7% of patients had a positive swab; in DTG, 19.9% had an early positive swab that was immediately treated by targeted antibiotics; and 16.7% had a mid-pregnancy positive swab. The DTG showed a significantly lower incidence of pPROM in univariate analysis (14.4% vs. 23.1%, *p* = 0.021), which was confirmed by multivariate analysis (OR 0.55, CI 0.33–0.93, *p* = 0.025). **Conclusions**: Our study suggests that, in asymptomatic women with twin pregnancy, the early screening of LGTIs and bacterial vaginosis by a cervico-vaginal swab at 12–14 weeks of gestational age is effective in reducing the risk of pPROM.

## 1. Introduction

The incidence of twin pregnancy has been rising in recent decades, mainly due to an advanced maternal age and the increased widespread availability of Medically Assisted Reproduction (MAR) [1,2]. Twin pregnancy is associated with an increased risk of maternal and perinatal mortality and morbidity [3]. In particular, preterm birth (commonly defined as birth after the time of viability, but before 37 weeks of gestational age) represents the main cause of adverse outcomes in newborn twins. Because of prematurity, the risk of neonatal mortality in multiple pregnancies increases four-fold compared to singleton pregnancies. Moreover, among twins, more than half of neonatal deaths are attributed to preterm birth [4,5,6].

Preterm birth in twin pregnancy occurs in about 59% of cases before 37 weeks of gestational age (compared to 11% in singleton pregnancies), and in 11% of cases before 32 weeks of gestational age (compared to 2% in singleton pregnancies) [7]. Preterm newborns have a higher incidence of complications, with severe neonatal morbidity, mortality and neuro-behavioral issues in the long term (i.e., cerebral palsy, visual and hearing impairment) [8,9,10,11,12,13,14,15,16,17].

Most preterm deliveries in twin pregnancies result from either preterm premature rupture of membranes (pPROM) or spontaneous onset of labor [18]. However, one should not forget that a significant number of preterm births are iatrogenic: indeed, about one fourth of preterm deliveries in twin pregnancies are medically indicated due to maternal (hypertensive disorders of pregnancy, pre-eclampsia, or gestational cholestasis) or fetal indications (intrauterine fetal growth restriction, non-reassuring fetal monitoring, or monochorionic-related complications such as twin to twin transfusion syndrome) [4,19].

The etiology of spontaneous preterm birth in twin pregnancy is most likely a combination of contributing factors. In singleton pregnancies, it has been reported that the onset of labor at a molecular level is mediated by the upregulation of specific proteins that promote cervical ripening and membrane rupture [4]. Such upregulation is normally determined by increased uterine stretch, the augmented production of corticotrophin-releasing hormone (CRH) by the placenta and the increased secretion of surfactant molecules by the fetal lung into the amniotic fluid. As Stock and colleagues reported, in multiple pregnancies, it is likely that these physiological stimuli are augmented due to the greater fetal size and larger placental and amniotic fluid volumes, leading to uterine overdistention and the increased secretion of CRH [4,20,21].

However, pathological processes can add up to these para-physiological mechanisms and contribute to an increased risk of premature labor; intrauterine infection or inflammation, uterine hypoxia/ischemia, placental insufficiency, cervical insufficiency, abnormal allograft reaction, stress, smoking, allergic phenomena and monochorionicity have been associated with preterm birth [4,6,21,22,23].

In particular, intrauterine infection has been documented in a considerable proportion of cases of preterm labor in multiple pregnancies. In a study by Romero and colleagues, amniotic fluid cultures tested positive in 11.9% of twin pregnancies that delivered prematurely [24]. Also, the amniotic sac of the presenting twin is the one most commonly affected by the infection, thus supporting the hypothesis of an ascending route [25]. 

It is known that lower genital tract infections (LGTIs) are related to preterm labor and birth in singleton pregnancies [4,5,23,24,25,26,27,28]. Moreover, recent studies have demonstrated that specific alterations in the vaginal microbiome, particularly certain pathogenic bacteria and a decrease in the richness and diversity of the vaginal microbial community, are significantly associated with preterm birth [29,30,31]. Twin pregnancy has also been associated with decreased microbiome diversity, thus suggesting a potential predisposing mechanism to LGTIs [32].

Considering the increased risk of spontaneous preterm births in twin pregnancy, it is crucial to identify successful screening methods and subsequent treatments to prevent prematurity. So far, only a few studies have evaluated the clinical application of a screening for LGTIs in asymptomatic women with twin pregnancy, and none of them could find a significant association between bacterial vaginosis at 22–34 weeks and preterm birth [33,34,35,36]. However, all these studies were carried out to detect the cervico-vaginal flora after 22–23 weeks of gestation, and not during early pregnancy. The aim of the present study was to assess whether the early identification and prompt treatment of bacterial vaginosis or other infections (such as Ureaplasma *spp*, Mycoplasma *spp*, Trichomonas vaginalis, and Gardnerella vaginalis) in asymptomatic women during the first trimester of a twin pregnancy could reduce miscarriages, the preterm premature rupture of membranes (p-PROM), and preterm birth. 

## 2. Materials and Methods

This is a retrospective cohort study. The study includes patients with twin pregnancies followed up at the Twin Pregnancy Care Unit of Sant’Anna Women’s Health Hospital in Turin (Italy) between September 2012 and February 2021. The pregnancies included in the study were referred to the Twin Pregnancy Care Unit within 12 weeks of gestational age. Pregnancies obtained using infertility medical treatments (ovulation induction) and/or a MAR (intrauterine insemination, in vitro fertilization, intracitroplasmatic sperm injection) were not excluded. 

Chorionicity was diagnosed in the first trimester by applying standard ultrasonographic criteria (presence of a lambda sign or of a T sign, along with two separate amniotic cavities) [37,38], and was later confirmed by post-partum placental examination. In spontaneous pregnancies, the gestational age was calculated according to the last menstrual period and confirmed by the first trimester ultrasound; for IVF pregnancies, it was determined on the basis of the day of blastocyst transfer in the uterus.

Our sample included both monochorionic diamniotic and dichorionic diamniotic pregnancies; differences in chorionicity have been taken into account in the multivariate analysis. Monochorionic monoamniotic and high-order multiple pregnancies were excluded due to the additional confounding factors that increase the risk of preterm birth and due to the iatrogenic indication to preterm delivery. 

Following the diagnosis of twin pregnancy, the women received a thorough counseling about the specific characteristics of their pregnancy, the risk of maternal, fetal and neonatal complications, the follow-up protocol and the possibility of performing screening tests or invasive diagnosis for fetal aneuploidy. 

Clinical examinations were scheduled monthly; they included the measurement of blood pressure and the prescription of lab exams such as a blood count, urine test and culture, serology for main prenatal infections and an oral glucose tolerance test (OGTT) at 24–28 weeks. Ultrasound scans were scheduled according to chorionicity, following the main international guidelines [37,38].

All twin pregnancies in our unit were managed by the same team of obstetricians and by applying a specific clinical protocol, in order to avoid any possible bias resulting from differences in the clinical management. Ultrasonographic assessments may have changed over time according to updates to the fetal surveillance guidelines [37,38,39,40], but the clinical follow-up protocol remained unchanged throughout the whole study period, since it is specific to our Centre.

Delivery was planned between 36 and 36^+6^ weeks of gestational age in the case of monochorionic diamniotic pregnancy, whereas it was scheduled between 37 and 37^+6^ weeks of gestational age in dichorionic pregnancy. A course of antenatal corticosteroids for lung maturation was offered to patients undergoing elective cesarean deliveries.

Women attending the clinic between 2012 and 2016 were submitted for a single cervico-vaginal swab at 20–22 weeks (Single-Test Group, STG), whereas women attending the clinic between 2017 and 2021 underwent two cervico-vaginal swabs, the first at 12–14 weeks and the second at 20–22 weeks (Double Test Group, DTG). After the direct visualization of the cervix with the use of a speculum, the cervico-vaginal swab was performed by taking two samples: an endocervical sample and a vaginal sample at the posterior fornix. Bacterial vaginosis was defined according to the Amsel criteria [41]: (a) vaginal pH > 4.5 as a consequence of decreased lactobacillary flora; (b) the presence of Clue cells on fresh microscopic examination; (c) homogeneous whitish or greenish greyish leucorrhoea, attached to the vaginal walls; and (d) a positive whiff-test, due to the release of amines following the addition of a drop of KOH to the vaginal secretion. Women with a positive cervico-vaginal swab were immediately treated with adequate antibiotic therapy: in the case of Ureaplasma or Mycoplasma *spp* infection, oral azithromycin (500 mg/die for six days) or clindamycin (300 mg twice a day for six days) was administered, whereas Trichomonas, Gardnerella, and bacterial vaginosis were treated with oral metronidazole (500 mg twice a day for seven days) or clindamycin. The screening protocol for LGTIs, the definition of bacterial vaginosis and antibiotic treatments did not change over the time period of the study.

For both STG and DTG, the following data were collected: maternal age, body mass index (BMI), parity, ethnicity, mode of conception (spontaneous or by MAR), chorionicity, moderate preterm birth (within 34 weeks), very preterm birth (within 31 weeks), extremely preterm birth (within 28 weeks), and late spontaneous abortion (within 24 weeks). 

The results are shown as an absolute value and relative frequencies (%) for categorical data, and as mean ± SD for continuous variables. We compared the STG and DTG for the rate of miscarriage, pPROM, and spontaneous preterm birth at ≤28, ≤31, and ≤34 weeks. In the univariate analysis, both a chi-square test (categorical variables) and t test (continuous variables) were carried out. Multivariable analysis models were then fitted to investigate the independent effects of a single vs. double swab on each outcome, which was set as a dichotomous variable. Miscarriages, pPROM and preterm birth at ≤28, ≤31 and ≤34 weeks were set as dependent variables; the group (STG or DTG), maternal age, parity, invasive prenatal tests, chorionicity, artificial conception and ethnicity were considered independent variables for the miscarriage, preterm birth and pPROM models. For all tests, a *p*-value < 0.05 was considered significant. All analyses were performed with Stata 14 software.

Because of the anonymous data collection and its retrospective nature, this study was deemed exempt from approval by the local review board. It fully adheres to the World Medical Association Declaration of Helsinki (as revised in 2013) and complies with the ethical standards of national and institutional committees on human experimentation. Written informed consent for the use of personal information was obtained from every participant using a designated form that was signed at the time of the first visit.

## 3. Results

From September 2012 to February 2021, 1177 twin pregnancies were referred to the Twin Pregnancy Care Unit. High-order multiple pregnancies (*n* = 102), monochorionic-monoamniotic pregnancies (*n* = 24), and twin pregnancies first observed after a gestational age of 12 weeks (*n* = 512) were excluded from the study. A total number of 539 monochorionic diamniotic or dichorionic diamniotic twin pregnancies were recruited in the study, among which 43 had a miscarriage in the first trimester and 13 were complicated by twin-to-twin transfusion syndrome (TTTS). 

A final cohort of 476 women was included in the analysis (of which 327 were dichorionic diamniotic). Among them, 285 underwent a single cervico-vaginal swab at 20–22 weeks (Single Test Group, STG), whereas 191 received two tests, one at 12–14 weeks and another at 20–22 weeks (Double Test Group, DTG). 

In the STG, 62 women (21.7%) had a positive swab; in the DTG, 38 women (19.9%) had a positive swab at 12–14 weeks and the other 32 (16.7%) had a positive swab at 20–22 weeks. Among these 32 women with a positive second swab, 17 were negative at the first one, whilst 15 were already positive and were treated, with another positive result at the second swab.

As illustrated in Table 1, the STG and DTG did not differ in their pre-gravidic BMI, non-white ethnicity, incidence of gestational diabetes and hypertensive disorders. The DTG included significantly more pregnancies obtained by MAR, dichorionic twins, and nulliparous women; also, maternal age in the DTG was significantly higher than in the STG. Gestational age at birth, rate of labor induction and rate of cesarean delivery were not significantly different between the two groups, whilst a significant difference in the use of invasive prenatal tests (19.6% in STG vs. 10.2% in DTG) was registered.

As shown in Table 2 (univariate analysis), a comparable rate of miscarriage occurred in the two groups: 4.6% in the STG and 5.2% in the DTG, respectively. In contrast, a significantly lower incidence of pPROM was observed in the DTG (14.4% vs. 23.1%, *p* = 0.021). In detail, among the women in the DTG, no significant difference was found in the rate of pPROM between those who had a negative result and those who, after a positive result, underwent antibiotic therapy; similarly, in the STG, no difference was found in the rate of pPROM among the negative women vs. positive women who were treated (not shown). We observed a comparable incidence of preterm delivery within 28 weeks (2.2% in DTG vs. 4.8% in STG), 31 weeks (9.9% in DTG vs. 9.3% in STG), and 34 weeks (32.5% in DTG vs. 30.1% in STG).

In the multivariate analysis (Table 3 and Table 4), none of the variables considered in the model (group, maternal age, parity, chorionicity, invasive prenatal tests, mode of conception and ethnicity) significantly affected the overall risk of miscarriage or preterm birth. Women in the DTG had a significantly lower risk of pPROM (OR 0.55 [0.33; 0.93]; *p* = 0.025), whilst maternal age, chorionicity and having undergone an invasive prenatal test did not significantly affect the overall risk of pPROM. Parity was significantly associated with the risk of pPROM in the multivariate analysis.

## 4. Discussion

The incidence of premature birth in our sample is comparable to that reported in the literature, with about one third of women giving birth before 34 weeks of gestational age [21,33,42].

Several screening tools for predicting the risk of preterm birth have been proposed by the scientific community, mainly cervical length assessment, fetal fibronectin assessment, home uterine activity monitoring, amniotic fluid sludge, and digital cervical assessment [21,33]. However, none of these methods have been validated by clinical practice guidelines as an effective screening for preterm birth in multiple pregnancies [40]. Surely, the lack of effective treatments to prevent preterm labor make it even more challenging to demonstrate improvements in perinatal outcomes, even if the correct screening tool had indeed been found. 

Actually, a number of different preventive methods and treatments have also been proposed to prevent preterm birth: bed rest, progesterone administration (vaginal, oral, intramuscular), cervical cerclage and cervical pessary; however, none of these seem to be effective or show conflicting results in twin pregnancy [33,42,43]. In particular, among singleton pregnancies, progesterone has shown promising results, which still have not been confirmed in multiple pregnancies [44]. 

A recent study concluded that the transvaginal assessment of the cervical length before 24 weeks of gestation remains the best tool for the prediction of spontaneous preterm delivery in women with twin pregnancy, though it did not consider screening for infection among the possible options [21]. 

The idea that lower genital tract infections may be associated with preterm birth is supported by the findings that specific alterations in the vaginal microbiome, particularly certain pathogenic bacteria and a decrease in the richness and diversity of the vaginal microbial community, are significantly associated with preterm birth [29,30,31]. 

Screening for lower genital tract infections has the potential to truly prevent a significant number of preterm births, since it is associated with an effective treatment consisting of antibiotics and probiotics that can eliminate pathological bacteria and restore the appropriate biodiversity of the vaginal microbiome. This consideration differentiates such an approach from all others, especially considering that other available treatments (progesterone, cervical pessary or cervical cerclage) seem to be much less effective in multiple pregnancies compared to singleton pregnancies.

Previous studies regarding singleton pregnancies at risk of preterm birth reported that the early screening and treatment of LGTIs could reduce the incidence of pPROM, preterm birth and miscarriage [45,46]. Similarly, the efficacy of immediate antibiotic treatment in the case of asymptomatic bacteriuria was reported to decrease the incidence of pyelonephritis and preterm birth [47]. A recent study showed that a short cervix is associated with an altered vaginal microbiome community structure in singleton pregnancies; also, this paper reported that the cervical cerclage is associated with an alteration in the vaginal microbiome (i.e., microbial community harboring a relatively low abundance of Lactobacillus), which was associated with a higher risk of preterm labor episodes, and with adverse outcomes [48].

Rather surprisingly, the currently available data about twin pregnancies do not allow conclusions to be drawn regarding the idea that early screening for cervico-vaginal infections and the prompt treatment of LGTIs can lower the incidence of obstetrical complications that lead to premature birth. To date, only a few studies have investigated the possibility of performing a screening for bacterial vaginosis and other lower genital tract infections in twin pregnancy [34,35,36,49]. American, French and English guidelines do not include a screening for LGTIs in their protocols for the management of twin gestation [40,50,51]. One review attributed a modest predictive value to bacterial vaginosis for preterm birth in twin pregnancy, refraining from recommending the early screening of LGTIs [5]. These conclusions, however, come from a limited number of studies that involved an overall low number of patients; namely, Goldenberg [34] recruited 147 patients, Wennerholm [35] recruited 121, and Ruiz [36] recruited 48 patients. Furthermore, in all previous studies, LGTIs screening was always performed after 22 weeks of gestational age (between 24–28 weeks in the first two studies and 22–34 in the third one). This does not allow the possible effect of early antibiotic treatment (before 20 weeks) in the prevention of preterm birth, which has already been shown in singleton pregnancies, to be evaluated [45,46]. Therefore, a possible explanation for the lack of efficacy found in LGTI screening by these studies is the fact that an appropriate treatment was administered much later in pregnancy, possibly when the pathological alterations mediated by infection and inflammation in the cervix had already been established. Our data suggest that the early screening of LGTIs in the first trimester may be more effective in reducing the rate of pPROM in twin pregnancy, possibly because the prompt treatment of infections and vaginal flora alterations allows for a timely improvement.

In the present study, the continuity of assistance offered by the same group of specialists allowed for good consistency in the clinical follow-up and treatment of obstetric complications. It should also be noted that there are, to this day, few specific guidelines on the clinical management of multiple pregnancies (mainly NICE and ACOG practice guidelines [39,40]), as most other guidelines focus mainly on fetal ultrasonographic surveillance. Therefore, the protocol for the clinical management of maternal complications in twin pregnancies did not change over the whole time period considered in this study (2012–2021), because it is specific to our Centre.

The two groups of patients (STG and DTG) showed significant differences in important population characteristics, such as a lower proportion of spontaneous pregnancies and monochorionic twins, a higher maternal age and a higher rate of nulliparous women in the DTG. All these could potentially be confounding factors. Therefore, they have been included in the multivariate analysis: all these factors, except for parity, were not found to be significantly associated and thus likely had a very limited impact on the results. On the contrary, parity and belonging to the STG or DTG both maintained significance in the multivariate model. We can therefore conclude that the association between the DTG and the reduction in pPROM is indeed confirmed. Moreover, parity is not a recognized risk factor for preterm birth [52].

It is rather surprising that our data showed no association between chorionicity and the risk of preterm birth: this finding is in disagreement with what was reported by Marleen and colleagues, who found a two-fold increase in the incidence of preterm birth before 28 weeks in monochorionic twins compared to dichorionic ones. However, in this meta-analysis, most studies included data on both spontaneous and iatrogenic preterm delivery. Since monochorionic twins present with specific complications because of their shared placenta (twin-to-twin transfusion syndrome, selective intrauterine growth restriction, and twin anemia polycythemia sequence), the increased rate of preterm birth could be due to the quota of complicated pregnancies that required delivery for maternal or fetal indications. Indeed, in our study, only women with pPROM or preterm labor were included, and this may explain the differences in the results. Our data are more in agreement with the study conducted by Carter and colleagues [53], who reported that the rates of preterm labor and the preterm premature rupture of membranes were not significantly different in dichorionic versus monochorionic pregnancies; as could be expected, this group confirmed that monochorionic twins are delivered at an earlier gestational age than dichorionic twins (34.2 weeks vs. 35.0 weeks), but such a difference was due to iatrogenic preterm birth and not to spontaneous labor or pPROM.

The limitations of the current study include its retrospective nature, which does not allow definite conclusions on the matter to be drawn, the rather long time period and the potential confounding effect of parity in our dataset. Chorionicity, the maternal age, ethnicity and invasive prenatal testing did not contribute significantly to preterm birth or pPROM in the multivariate analysis, thus allowing us to exclude their confounding effect. Another limitation that should be considered is the lack of information regarding the anamnestic risk of women included in the study, i.e., history of previous preterm birth or previous cervical LEEP, which have been confirmed as risk factors for preterm birth [54]; this information could be extremely useful when aiming to control the possible confounding effect of potential differences between the two groups. Finally, one limitation of the study is the lack of information on the perinatal and long-term outcomes of newborns, including their neurological development; a 2-year period of follow up could be needed to investigate whether the reduction in pPROM is related, independent of gestational age at birth, to better neonatal outcomes. 

With the limitations of a retrospective analysis, the present study is one of the first clearly showing that the early screening of LGTIs and treatment of LGTIs in the first trimester is significantly more effective for the prevention of pPROM than performing a single swab at 20–22 weeks. In fact, patients with a positive cervico-vaginal swab during the first trimester could be treated earlier with targeted antibiotics. Also, there was no difference in the rate of pPROM between those who had a negative result and those who, after a positive result, received antibiotic therapy. This result further supports the idea that the early screening and treatment of LGTIs in asymptomatic women with a twin pregnancy could reduce the risk of pPROM and, possibly, of preterm birth. We also observed a trend toward a decreased rate of preterm births (irrespective of the gestational age) in patients who underwent the first trimester cervico-vaginal swab; the lack of statistical significance could be due to the sample size or to the presence of other confounding risk factors. This indicates the need to carry out further, larger studies. 

In conclusion, our study suggests that, in asymptomatic women with a twin pregnancy, the early screening of LGTIs by cervico-vaginal swab during the first trimester could significantly lower the risk of pPROM. Whether this could be effective in reducing the incidence of preterm birth and improving the neonatal and infant long-term prognosis should be clarified by further, adequately powered studies.

## Figures and Tables

**Table 1:** Main clinical and demographic characteristics of the women included in the study. Values are expressed as mean ± SD or as absolute value and percentage, as appropriate.

	STG (*n* = 285)	DTG (*n* = 191)	*p*-Value
Maternal age (years)	32.4 ± 5.2	34.2 ± 5.1	**<0.001**
Pre-gravidic BMI ^1^ (kg/sqm)	23.2 ± 4.3	22.7 ± 3.9	0.177
Non-white ethnic group	81 (28.4)	47 (24.6)	0.358
Nulliparous	169 (59.3)	133 (69.6)	**0.022**
Dichorionic-diamniotic	185 (64.9)	142 (74.3)	**0.030**
Artificial conception	77 (27.0)	80 (41.9)	**0.001**
GDM ^1^	41 (14.9)	41 (21.7)	0.062
Hypertensive disorders	38 (13.9)	26 (13.7)	0.973
Invasive prenatal testing	56 (19.9)	19 (10.2)	**0.005**
Gestational age at birth	34.1 ± 4.05	34.4 ± 3.71	0.272
Cesarean section	208 (75.9)	146 (78.9)	0.622
Elective cesarean section	160 (58.4)	105 (56.8)	0.600
Induced labor	42 (15.3)	29 (15.7)	0.888

^1^ BMI, Body Mass Index; GDM, gestational diabetes mellitus.

**Table 2:** Miscarriages, preterm premature rupture of membranes (p-PROM) and preterm births: results of the univariate analysis. Values are expressed as an absolute value and percentage.

	STG (*n* = 285)	DTG (*n* = 191)	*p*-Value
Late miscarriages	13 (4.6%)	10 (5.2%)	0.737
pPROM ^1^	64 (23.1%)	27 (14.4%)	**0.021**
Preterm birth at ≤28 weeks GA ^1^	13 (4.8%)	4 (2.2%)	0.153
Preterm birth at ≤31 weeks GA ^1^	27 (9.9%)	17 (9.3%)	0.831
Preterm birth at ≤34 weeks GA ^1^	89 (32.5%)	55 (30.1%)	0.584

^1^ pPROM, preterm premature rupture of membranes; GA, gestational age.

**Table 3:** Results of the multivariate analysis for late miscarriage and pPROM. OR = odd ratio; CI 95% = confidence interval.

	Late Miscarriage		pPROM	
	OR (95% CI)	*p*-Value	OR (95% CI)	*p*-Value
Group (STG or DTG) ^1^	1.58 (0.65–3.98)	0.350	**0.58 (0.43–0.92)**	**0.024**
Maternal age	1.1 (0.92–1.12)	0.753	1.02 (0.97–1.06)	0.414
Parity	0.92 (0.35–2.41)	0.869	**0.54 (0.32–0.91)**	**0.019**
Chorionicity	0.84 (0.67–1.15)	0.774	0.76 (0.42–1.34)	0.238
Invasive prenatal tests	0.80 (0.18–3.71)	0.768	1.21 (0.65–2.27)	0.459
Ethnicity	2.38 (0.90–6.20)	0.068	1.57 (0.43–4.65)	0.384
Artificial conception	1.35 (0.96–2.65)	0.072	1.14 (0.62–1.43)	0.143

^1^ STG, single test group; DTG, double test group.

**Table 4:** Results of the multivariate analysis for preterm birth. Results are shown as OR (CI 95%). OR = odd ratio; CI 95% = confidence interval.

	Preterm Birth		
	≤28 Weeks	≤31 Weeks	≤34 Weeks
Group (STG or DTG) ^1^	0.45 (0.14–1.44)	1.04 (0.53–2.05)	0.97 (0.63–1.48)
Maternal age	0.96 (0.87–1.06)	0.94 (0.85–1.11)	1.00 (0.96–1.04)
Parity	0.94 (0.44–2.65)	0.85 (0.64–1.74)	1.04 (0.69–1.59)
Chorionicity	0.58 (0.18–1.85)	0.71 (0.34–1.45)	1.21 (0.79–1.86)
Invasive prenatal tests	0.97 (0.64–1.78)	0.84 (0.30–2.31)	1.08 (0.61–1.90)
Ethnicity	1.37 (0.48–4.12)	1.23 (0.60–2.51)	1.11 (0.70–1.78)
Artificial conception	1.48 (0.76–2.87)	1.24 (0.45–2.34)	1.12 (0.84–3.12)

^1^ STG, single test group; DTG, double test group.

## Data Availability

The data presented in this study are available upon request from the corresponding author. The data are not publicly available due to privacy reasons.
